# Third Director of the European Centre for Disease Prevention and Control takes office

**DOI:** 10.2807/1560-7917.ES.2017.22.25.30560

**Published:** 2017-06-22

**Authors:** Eurosurveillance editorial team

**Affiliations:** 1 European Centre for Disease Prevention and Control, Stockholm, Sweden

On Friday 16 June 2017, Dr Andrea Ammon took up office as the third Director of the European Centre for Disease Prevention and Control (ECDC) following her election by the Centre’s management board earlier the same year. The appointment follows a two-year tenure as acting director during which Dr Ammon steered the ECDC steadily and calmly through a challenging period when besides the Centre’s day-to-day work, expertise and resources were requested for the European preparedness and response to global threats such as the Ebola and Zika virus disease outbreaks in Africa and the Americas [1,2].

Dr Ammon joined the newly established ECDC already in May 2005, as one of its first employees and Head of the Surveillance Unit [3]. While still setting up the unit, she was instrumental in drafting and implementing a long-term surveillance strategy for the European Union (EU). As part of this, she and her enthusiastic team evaluated the existing 17 European Dedicated Surveillance Networks (DSN) which included well-established and widely known networks such as EURO TB and EURO HIV, and gradually transferred them into the ECDC [4]. In parallel, her unit developed The European Surveillance System (TESSy), revised the EU case definitions and produced for the first time an Annual Epidemiological Report on infectious diseases in the EU.

From April 2011 to April 2015, Andrea Ammon was Deputy to the Director and ECDC’s Head of Unit for Resource Management and Coordination. She was a member of the scientific committee for the European Scientific Conference on Applied Infectious Disease Epidemiology (ESCAIDE) and in 2014 and 2015, she headed the committee.

A medical doctor by training, Dr Ammon discovered her passion for public health early in her career and she has extensive experience in working in public health authorities at differing levels. Starting at the local and then regional level in the German federal state of Bavaria, she moved to the national public health institute, the Robert Koch Institute (RKI) in 1996, where she was among the first national Field Epidemiology Programme trainees and simultaneously a member of the first cohort of the European Programme for Intervention Epidemiology Training (EPIET). At RKI, she became the Head of Department for Infectious Disease Epidemiology and State Epidemiologist for Germany from late 2002 to 2005. Besides coordinating the national outbreak response team for current and emerging infections, she directed the national field epidemiology training programme and coordinated emergency planning for influenza and epidemiological research programmes in infectious diseases. Furthermore, she provided scientific advice for government ministries, Members of Parliament and the public. In 2003, she coordinated the German response to Europe’s first imported case of severe acute respiratory syndrome (SARS). During her time at RKI, Dr Ammon also became a nationally and internationally respected expert in the field of food- and waterborne diseases. Her PhD was on the synergy between epidemiology and microbiology in the prevention and control of food-borne diseases.

Dr Ammon’s professional and leadership skills are complemented by other strong characteristics such as a mind open to suggestions, a capacity for motivating staff and an acute sense of fairness.

The *Eurosurveillance* journal and its editors benefited from Dr Ammon’s strategic vision and sense for quality between 2007 and 2015, when she was an associate editor and a strong supporter of the journal. She resigned from this position when taking up her post as ECDC Director to mark the editorial independence of the journal from its publisher and its Director.
